# Recent Bioinformatic Progress to Identify Epigenetic Changes Associated to Transposable Elements

**DOI:** 10.3389/fgene.2022.891194

**Published:** 2022-05-13

**Authors:** Emmanuelle Lerat

**Affiliations:** Univ Lyon, Univ Lyon 1, CNRS, VetAgro Sup, UMR5558, Laboratoire de Biométrie et Biologie Evolutive, Villeurbanne, France

**Keywords:** epigenetics, transposable elements, bioinformatics, epigenomics, NGS data

## Abstract

Transposable elements (TEs) are recognized for their great impact on the functioning and evolution of their host genomes. They are associated to various deleterious effects, which has led to the evolution of regulatory epigenetic mechanisms to control their activity. Despite these negative effects, TEs are also important actors in the evolution of genomes by promoting genetic diversity and new regulatory elements. Consequently, it is important to study the epigenetic modifications associated to TEs especially at a locus-specific level to determine their individual influence on gene functioning. To this aim, this short review presents the current bioinformatic tools to achieve this task.

## Introduction

For many years, the presence of transposable elements (TEs) has been acknowledged in the genomes of all living organisms, not only because of their large prevalence in some of them but also because they have a profound impact on their functioning and evolution (Feschotte, 2008; Biemont, 2010; Almojil et al., 2021). TEs correspond to sequences with a large variety of structural features allowing their grouping in different classes and families (Wicker et al., 2007). They share the common characteristics to be able to move around inside their host genome using their own enzymatic machinery and to duplicate themselves, leading them to be present in a genome in multiple and often very similar copies. The presence and expression of TEs have been associated to various diseases like for example the Haemophilia A, the Alstrom syndrome, the Dent’s disease or different cancers [see for a review (Payer and Burns, 2019)]. Their mutational effects are generally summarized into three categories: the disruption or modification of a host protein, the deregulation of gene transcription, and chromosomal rearrangements by ectopic recombination.

The deleterious effects of TEs on their host genomes have led to the evolution of regulatory mechanisms to control their activity. In particular, the different epigenetic mechanisms contribute to their silencing preventing their mobilization (Huda and Jordan, 2009; Lisch, 2009). In mammals and in plants, TEs are mainly silenced by DNA methylation (Deniz et al., 2019). TEs are also targeted by repressive histone modifications leading to heterochromatin formation (Slotkin and Martienssen, 2007). Finally, RNA interference through the action of small RNAs (sRNAs) allows post-transcriptional silencing leading for example to the control of TE activity in the germline of *Drosophila melanogaster* (Sato and Siomi, 2020). sRNAs also allow the targeting of TEs by DNA methylation of homologous sequences and the setting up of histone modification to silence them, which demonstrate the clear interlacing of all epigenetic mechanisms (Hall et al., 2002; Lewsey et al., 2016).

Despite their numerous deleterious effects, TEs are also now largely recognized as important actors in the evolution of genomes by promoting a fair amount of genetic diversity (Schrader and Schmitz, 2019). In addition, TEs can be implicated in the gene regulation by providing regulatory elements (Chuong et al., 2016). The genetic diversity induced by TEs is particularly valuable for organisms in order for them to adapt to environmental changes. This new response may be very rapid especially when it involves epigenetic changes associated to TEs that may induce an impact on the host gene functioning. For example, a specific TE insertion in *D. melanogaster* was shown to display inactive histone modifications in normal condition but also active modifications under oxidative stress conditions, having an impact on the observed expression pattern of nearby genes (Guio et al., 2018). Similarly in mouse, the methylation level of a TE inserted near the agouti gene leads to variation in its transcription level inducing variation in the fur color (Morgan et al., 1999). In some cases, the TE associated epigenetic modifications may act as facilitator for adaptation and may be conserved trough time corresponding to the taming of TEs (Capy, 2021). In addition, some TEs may be organized in genomes as tandem arrays which has been proposed to participate to chromosome rearrangement and structuring in some plants (Kalendar et al., 2020).

Consequently, it is particularly important to study the epigenetic modifications associated to TEs as already proposed elsewhere (Lerat et al., 2019). More particularly, it is even more important to have information at the copy specific level of TEs, i.e. according to the insertion locus of each element. Indeed, it can be expected that not all copies of a given TE in a genome may have the same impact on genes. Although fixed TE insertions are ancient and likely to be not deleterious in normal condition, they may influence their neighboring genes when a change in the environment occurs (Grégoire et al., 2016). In genomes, there is also a quite large proportion of TE insertions that are polymorphic when comparing different individuals of a same species (Gardner et al., 2017). These insertions can represent a potential source of genetic variability between individuals (Goubert et al., 2020). A very large number of bioinformatic tools have been designed to identify polymorphic insertions using genomic re-sequencing data by comparison to a reference genome. Some of these tools have been developed to identify insertions of interest in specific diseases like cancer, in which TEs have been described for a long time to be reactivated potentially leading to new insertions (Anwar et al., 2017; Jang et al., 2019). Among the twenty existing programs (for a comprehensive list, see https://tehub.org/en/resources/repeat_tools), we can cite for the most recent *TIP_finder* (Orozco-Arias et al., 2020) developed to detect TE insertions in very large genomes and tested on human cancer data, *cloudMELT* (Chuang et al., 2021) which was used to discover thousand of polymorphic TE insertions in numerous human population data, and *xTea* (Chu et al., 2021) which identifies new TE insertions from multiple sequencing technologies including long-reads and 10X linked reads. Although some attempts to perform standardized benchmarks of these methods exist (Rishishwar et al., 2017; Vendrell-Mir et al., 2019), there is nevertheless still room to determine what is the best tool to use considering the investigated biological data.

**FIGURE 1:**
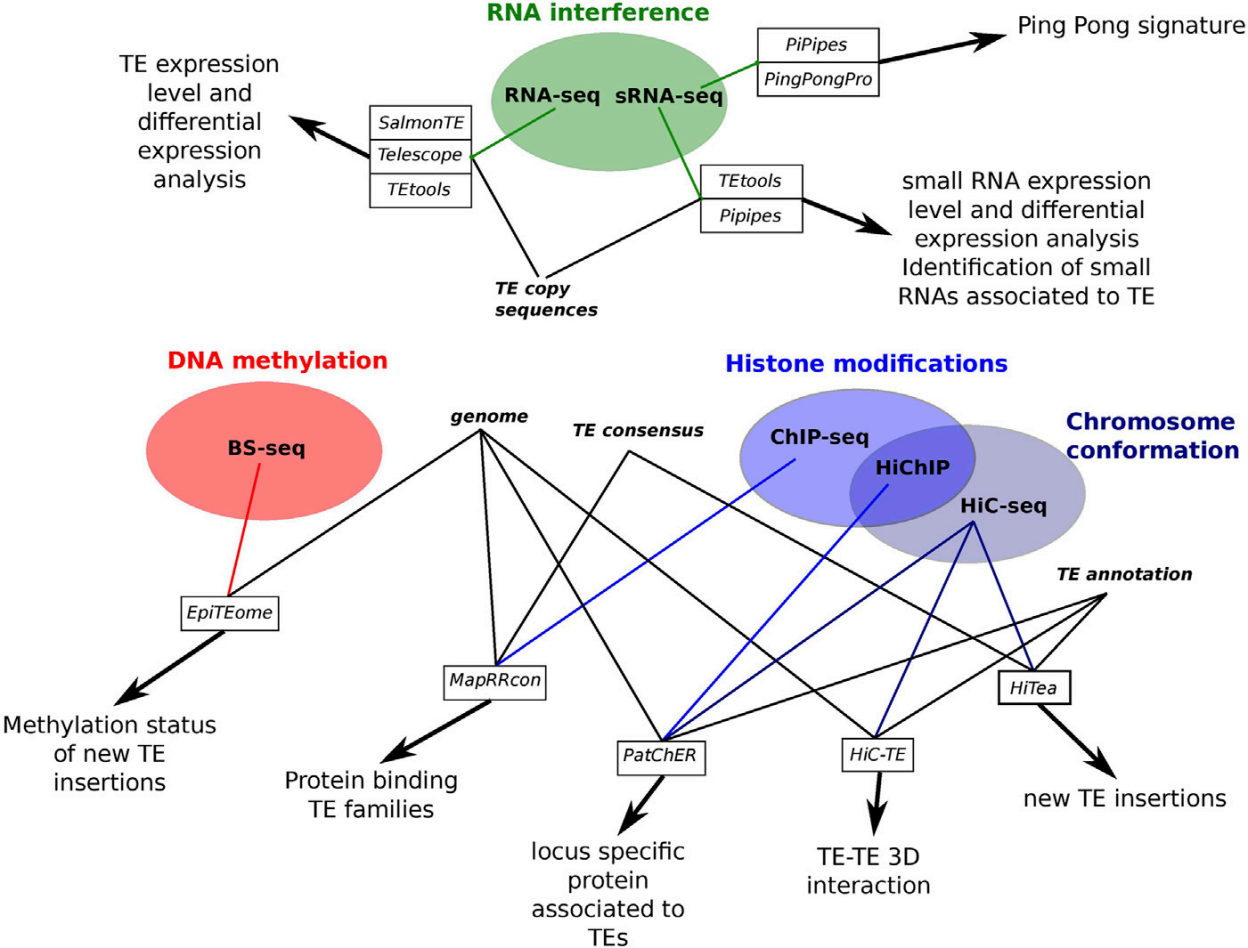
Summary scheme of the different tools performing epigenetic analysis associated to TEs presented in this review.

The question of the locus specificity in the epigenetic analysis of TEs is very difficult to tackle on the bioinformatic point of view given the sequencing data obtained by the different techniques used to generate them. Contrary to whole genome re-sequencing, it is more difficult to obtain reads long enough to allow unambiguous mapping of reads on the reference genome, especially in the case of histone modification analysis. This comes from the fact that recent TE copies are very similar in sequences and that short reads may not be specific enough to distinguish between very similar TE copies. Thus, the question of the sequenced read mappability is central in the development of bioinformatics tools aimed to study the epigenetic modifications associated to TEs. Indeed, short reads corresponding to TEs are often difficult to map unambiguously because of the repeated nature of TEs and the high intra-family sequence similarity (Li and Freudenberg, 2014; Sexton and Han, 2019; Teissandier et al., 2019). In this mini-review, I will present the most recent bioinformatic tools that have been proposed to decipher epigenetic modifications specifically associated to TEs and trying to take into account the copy specific level of these insertions (Figure 1).

## Transcription and RNA Interference Analyses

A first indication concerning the activity of TEs and thus the potentiality for them to either promote new insertions or influence neighboring gene expression, is the presence of TE transcripts in the cells. Since few years, specific bioinformatic tools have been developed to measure the transcription level of TEs (Table 1), some also allowing their differential expression analyses among several samples (different tissues or different populations). In their very comprehensive review concerning this topics, Lanciano and Cristofari (Lanciano and Cristofari, 2020) perfectly describe the complexity of the task. Indeed, since TEs are repeats that can be polymorphic, highly similar and with potential overlapping with genes, it drives to the existence of complex transcripts which may be difficult to identify and analyze. The authors identify three challenges to be overcome to deal with TE expression analysis which are the mappability of reads corresponding to TEs which are often of non-unique location, the sequence divergence among copies from a same family and their chimeric or co-transcription with genes blurring the detection of functional TE transcripts (Lanciano and Cristofari, 2020). In an attempt to determine how existing tools can accurately assess the expression of TEs at the copy level, a simulation study was recently performed (Schwarz et al., 2021). In this study, RNA-seq reads from mouse, human and turquoise killifish were generated. The results of this benchmark indicate that three of the tested tools performed very well to detect differentially expressed TEs. The first tool giving the best performance is *SalmonTE* (Jeong et al., 2018). It performs a read mapping against TE sequences and reassigns multi-mapping reads using the expectation-maximization (EM) algorithm before determining the read count of each sequence. The second best tool, *Telescope* (Bendall et al., 2019), was designed to determine TE expression in a copy-specific manner. As the previous tool, it uses EM algorithm to assign ambiguous mapped reads. Finally, the third position is occupied by *TEtools* (Lerat et al., 2017) which performs read alignment against TE sequences allowing transcription level determination at both family and copy level. Contrary to the other two tools, it randomly assesses multi-mapped reads to a given sequence. According to the benchmark, the three tools have the potential to precisely assess TE expression at a specific genomic locus with some minor modifications (Schwarz et al., 2021). Other tools continue to be developed with the goal to determine the genomic locus expression like for example *REdiscoverTE* (Kong et al., 2019) or *ExplorATE* (https://github.com/FemeniasM/ExplorATE_shell_script). The first tool takes advantage of the weight-mapping method *Salmon* (Patro et al., 2017) using *RepeatMasker* outputs and performs expression analysis down to the TE sub-family level. The quantification at individual locus level is accomplished with regard to the host gene positions i.e. whether the sub-family copies are intergenic, intronic or exonic. The second tool is an R package aims at identifying active TEs among RNA-seq data. It deals with both co-transcription and multi-mapping issues, and performs differential expression analyses. It may also be interesting to directly assess the effect of TEs on gene transcription. This is what is proposed using the R package *TEffectR* (Karakülah et al., 2019). This methods uses a linear regression model to determine the influence of TEs on neighboring gene expression using RNA-seq data, *RepeatMasker* output files containing TE location and Ensembl gene annotations. Similarly, the *LIONS* pipeline (Babaian et al., 2019) aims at identifying and analyzing the expression of chimeric TE-gene transcripts initiated by TEs.

**TABLE 1:** Recent tools for transcription and small RNA analysis.

Tool	Function	Input data	Algorithm for read mapping and selection	References
SalmonTE	Quantification of TE transcript abundance	RNA-seq and TE sequences	EM-algorithm	Jeong et al. (2018)
Telescope	Copy-specific TE expression	Aligned read from RNAseq	EM-algorithm	Bendall et al. (2019)
TEtools	Determine differentially expressed TEs and smallRNAs	SmallRNA-seq, RNA-seq, TE copies	Bowtie2 (mRNA) and Bowtie1 (smallRNA) (random assignment of best match)	Lerat et al. (2017)
REdiscoverTE	Copy-specific TE expression	RNA-seq, TE copy sequences, intron sequences	Salmon (EM-algorithm)	Kong et al. (2019)
ExplorATE	Copy-specific TE expression	Genome sequence, gene annotation, TE annotation RNA-seq	Salmon; target transcript assignation based on the percentage of identity for a class/family of TEs	https://github.com/FemeniasM/ExplorATE_shell_script
TEffectR	Influence of TEs on neighboring gene expression	Gene annotation, TE annotation, aligned RNA-seq reads on genome	Reads with 100% overlap with given TE regions are considered	Karakülah et al. (2019)
LIONS	Identification and analysis of chimeric TE-gene transcripts	RNA-seq, References genome, gene and TE annotation	Tophat2 (random assignment of best match)	Babaian et al. (2019)
PiPipes	Analyze piRNAs and TE-derived RNAs	SmallRNA-seq, RNA-seq, TE copies	STAR (mRNA) and Bowtie1 (smallRNA) (EM-algorithm)	Han et al. (2015)
PingPongPro	Detection of ping-pong cycle activity in piRNA-Seq data	SmallRNA-seq	Weighted read count	Uhrig and Klein, (2019)

Having the expression level of TEs is however not enough to evaluate whether a family is actually active and able to promote new insertions. To this aim, it is important to study the post-transcriptional regulation of TEs by analyzing the transcription of sRNAs, and more particularly of piwi-RNAs (piRNAs) which are specifically designed for the TE regulation. However, such a type of analyses comes with more technical difficulties. Reads corresponding to piRNAs are even shorter than those corresponding to mRNAs. Indeed, piRNAs have sizes comprised between 21 and 35 bp (Ozata et al., 2019), which usually requires the addition of a mononucleotide tail in order to obtain long enough fragments for high throughput sequencing. These tails need to be removed before the reads can be used, with for example a dedicated trimming tool like *UrQt* (Modolo and Lerat, 2015), designed at first for this task but also able to perform regular unsupervised trimming of any NGS data. To avoid as much as possible the presence of degraded mRNAs, it is usually necessary before sequencing to have a first step of filtering like the anion-exchange chromatography purification method (Grentzinger et al., 2014) which allows the enrichment of genuine piRNAs, resulting in an increase in their sequencing depth. Once the reads have been sequenced, the goal is to be able to correctly assign the piRNAs to their TE family as well as their expression level, to identify the TE families that are actively regulated. In that perspective, very few tools have been proposed to specifically handle this question (Table 1). The *TEtools* pipeline (Lerat et al., 2017) proposes such a possibility, using an alternative mapper than for mRNA to take into account the short size of the reads. One tool of the *piPipes* pipeline (Han et al., 2015) performs transcriptomic analyses of piRNAs based on the mapping of reads on consensus TE sequences from model species. In addition, it can report the “Ping-Pong” signature specific of the piRNAs, assigned to genomic annotations. This signature indicates the presence of the positive feedback loop (called the Ping-Pong cycle) allowing the reinforcement of piRNA production and thus of the TE-silencing effect. The detection of this particular signature is at the core of the *PingPongPro* tool (Uhrig and Klein, 2019) which uses weighted read count within the region of a TE overlapping by 10 bp and corresponding to the empirical probability that the read is ping-pong-derived.

## DNA Methylation and TE Insertions

DNA methylation has been described in numerous organisms as the main silencing mechanism of TEs at the transcriptional level on a long term (Deniz et al., 2019). In majority, the mechanism targets specifically 5-methylcytosine but other modifications exist like the 4-methylcytosine and the 6-methyladenine. Different techniques have been developed to decipher the methylation profile of a genome. The most widely used is the bisulfite sequencing (BS-seq) technique in which genomic DNA is sequenced after bisulfite conversion of unmethylated cytosines into uracils, methylated cytosines being protected from the conversion. The comparison with the sequence of the same genomic DNA without treatment allows to determine which cytosines in the genome are methylated in the studied condition. Currently, only one bioinformatic tool specifically address the question of the methylation of TE sequences. This tool, *EpiTEome* (Daron and Slotkin, 2017), aims at detecting both new TE insertion sites and their corresponding DNA methylation status. To achieve this goal, the first step is to use BS-seq data to identify new TE insertions when compared to a reference genome. Then the methylation status of these insertions is assessed as well as at the region surrounding them. The tool takes as input BS-seq reads that do not map on the reference genome using specific mappers designed to handle such data like *Bismark* (Krueger and Andrews, 2011). These reads are then split and each split-read is mapped on the reference genome to identify breakpoint locations on the read so that part of the read corresponds to a TE and the other part to a genomic sequence into which the new insertion occurred. The procedure allows the identification of all new TE insertion locations. The DNA methylation status is determined using the split-reads that were used to identify the insertion locus. The main drawback of the method is that it only considers insertions not present in the reference genome whereas they are not the only ones expected to be subject to methylation variation. This means that for these other insertions, it is still necessary to rely on classic approaches dealing by BS-seq data, knowing that they have limitations in their mapping abilities when it concerns TEs.

## Chromatin Structure and Chromosome-Chromosome Interactions

The DNA compaction inside the nucleus is associated to the various biochemical modifications directed on the amino-acid tail of the histone proteins. Some modifications are associated with the opening of the chromatin allowing gene expression whereas other modifications are on the contrary associated with closed chromatin (heterochromatin) which drives to transcription silencing (Lawrence et al., 2016). For example, the regulation of retroelements has been associated to the presence of trimethylated histone H3 at lysine 9 (H3K9m3) (Fukuda and Shinkai, 2020). On the contrary, histone acetylation is associated to gene expression (Verdone et al., 2005). Different bioinformatic tools have been developed to analyze the reads produced by Chromatin Immuno-Precipitation sequencing (ChIP-seq) (see for a review Steinhauser et al., 2016) but the majority of them discard multi-mapping reads preventing the identification of peaks associated to repeat regions. However, some recent tools do consider multi-mapping reads and thus may potentially give some information concerning repeat sequences. In that respect, among the most recent published tools, *Crunch* (Berger et al., 2019) considers multi-mapping reads to avoid a loss of binding peaks in repeated regions and equally distribute the weight of each of these reads to all mapping locations. Although this represents a good starting point, this is however not specific enough to allow the direct analysis of histone modifications associated to TEs.

There are very few tools that propose to assign histone modifications to TEs (Table 2). The first to have been developed estimates an average histone modification enrichment for a set of homologous repeat sequences, like a TE family, but does not give information about the variability among these sequences (Day et al., 2010). Moreover, among the multiple mapping reads on the genome, only those uniquely mapping to sequences belonging to a same repeat family are taken into account. This first tool is interesting but is also limited by the choice of organisms on which the analyses can be made. More recently, the pipeline *MapRRcon* was developed to allow the identification of proteins binding to TE sequences by mapping ChIP-seq reads to TE consensus sequences after whole-genome alignment (Sun et al., 2018). This approach was applied on human data to identify the interaction between transcription factors and a specific family of TE called L1, representing the youngest and most active TE family in human. Associated to transcriptomic analyses, it allowed the authors to suggest that some L1-binding factors may play a role in the regulation of L1 activity during tumor development. Again in this approach, the read assignment remains global and is only able to identify reads associated to the whole TE family without providing information concerning the genomic location of the individual insertions. More globally, a major problem with ChIP-seq reads in the identification of associated TE sequences is their short sizes which prevents obtaining a sufficient number of reads unambiguously mapped to individual TE locations. Very recently, a solution has been proposed to help bypass this difficulty which takes advantage of random 3D interactions as implemented in the pipeline *PatChER* (Taylor et al., 2021). These random interactions correspond to relatively short distance (few tens of kb) 3D folding of the chromosomes and can be obtained from high throughput technologies like HiC-seq. HiC-seq is a method allowing to decipher 3D architecture of whole genomes by coupling proximity-based ligation with high-throughput sequencing (Lieberman-Aiden et al., 2009). In their approach, Taylor et al. use the random 3D interaction to guide the mapping of short reads obtained from HiChIP, a protein-centric chromatin conformation method, on TE individual insertions. The principle of this method is that since HiC-seq produces chimeric fragments between genomic regions that are close in 3D space, it should thus be possible to pair non-unique reads with unique reads from nearby genomic regions when focusing on random 3D interactions. By applying their method on mouse embryonic stem cell data, the authors were able to show that a particular family of retrotransposon displays a large variation in the enrichment of an active histone modifications according to the genomic location of the TE insertions (Taylor et al., 2021).

**TABLE 2:** Recent tools for the identification of TEs in chromatin structure and chromosome-chromosome interactions.

Tool	Function	Input data	Multimapping handling	References
Crunch	Performs ChIP-seq analysis (mapping, peak calling)	ChIP-seq data and references genome	Multimapping reads are taken into account to avoid a loss of binding peaks in repeated regions; the weight of each of these reads are equally distributed to all mapping locations	Berger et al. (2019)
MapRRcon	Identify proteins binding to TE sequences	ChIP-seq data, references genome and TE consensus sequences	Unique and non-unique aligned reads are extracted and mapped to TE sequences; reads with partial alignment, >3 mismatches and any indels are excluded; Remaining reads are mapped against TE consensus	Sun et al. (2018)
PatChER	Use of chimeric HiC-seq fragments between unique and non-unique reads to identify proteins binding to TE sequences	HiChIP data, HiC-seq data, references genome	Performs random mapping of non-unique reads	Taylor et al. (2021)
HiC-TE	Identification of TEs implicated in 3D conformation	HiC-seq data, references genome	Read mapping performed using Bowtie2	Lexa et al. (2021)
HiTea	Identification of new TE insertions using discarded HiC-seq reads from classical approaches	HiC-seq data, TE consensus, TE annotations in references genome	Identification of close discordant read pairs with one mapping on a unique locus and the other on a TE sequence	Jain et al. (2021)

Chromosome interactions may also be interesting to study on the TE point of view. Since it has been shown that some TEs may be associated to particular 3D conformation (Cournac et al., 2016; Lu et al., 2021), they may be implicated in long distance gene regulation. Very recently, the pipeline *HiC-TE* (Lexa et al., 2021; https://gitlab.fi.muni.cz/lexa/hic-te) was proposed to study interactions in nuclei of different repeat sequences, and in particular TEs, in long distance or interchromosomal interactions. The tool identifies and quantifies the interactions of repeats in the 3D organization of the genome using as an input HiC-seq data. It can work according two modes, reference-based and reference-free, to enhance the robustness of the results. Using HiC-seq data can also allow the identification of new TE insertions as proposed by the tool *HiTea* (Jain et al., 2021). This approach uses reads that are discarded in classical analyses of HiC data and map them on TE consensus to identify non reference TE insertions. In their application, the authors used split HiC read information and coverage to detect insertions of three major classes of TEs active in human. They were able to detect 1,085 new insertions using Hi-C data from the GM12878 cell line, which represent 62% of the new insertions identified by whole genome re-sequencing using long reads.

## Conclusion

Advances in sequencing technologies have made it possible to develop new bioinformatic tools for more specific analysis of TEs. The most recent tools are beginning to be able to analyze in a finer way, i.e. at the level of the individual copy, the epigenetic modifications associated with them. However, there is still a lot of progress to be made in this field, in particular with the consideration of structural variants between the samples studied independently of a reference genome. Cellular heterogeneity within a tissue must also be taken into account, particularly in the case of cancer studies. For this last possibility, a new methodological development was made in this direction that allows the locus-specific expression analysis of TEs in single cells (Berrens et al., 2021). New bioinformatic tools should thus continuously be developed to help analyze these new types of data.
